# Diagnostic Value of the CSF α-Synuclein Real-Time Quaking-Induced Conversion Assay at the Prodromal MCI Stage of Dementia With Lewy Bodies

**DOI:** 10.1212/WNL.0000000000012438

**Published:** 2021-08-31

**Authors:** Marcello Rossi, Simone Baiardi, Charlotte E. Teunissen, Corinne Quadalti, Marleen van de Beek, Angela Mammana, Michelangelo Stanzani-Maserati, Wiesje M. Van der Flier, Luisa Sambati, Corrado Zenesini, Byron Caughey, Sabina Capellari, Afina W. Lemstra, Piero Parchi

**Affiliations:** From IRCCS (M.R., S.B., C.Q., A.M., M.S.-M., C.Z., S.C., P.P.), Istituto delle Scienze Neurologiche di Bologna; Department of Experimental, Diagnostic and Specialty Medicine (S.B., P.P.) and Department of Biomedical and Neuromotor Sciences (L.S., S.C.), University of Bologna, Italy; Neurochemistry Laboratory, Department of Clinical Chemistry (C.E.T.), and Department of Neurology (M.v.d.B., W.M.V.d.F., A.W.L.), Alzheimer Center Amsterdam, Amsterdam Neuroscience, Vrije Universiteit Amsterdam, Amsterdam UMC, the Netherlands; and LPVD (B.C.), Rocky Mountain Laboratories, National Institute of Allergy and Infectious Diseases, NIH, Hamilton, MT.

## Abstract

**Objective:**

To investigate whether the CSF α-synuclein (α-syn) real-time quaking-induced conversion (RT-QuIC) assay accurately identifies patients with mild cognitive impairment (MCI) due to probable Lewy body (LB) disease.

**Methods:**

We applied α-syn RT-QuIC to 289 CSF samples obtained from 2 independent cohorts, including 81 patients with probable MCI-LB (age 70.7 ± 6.6 years, 13.6% female, Mini-Mental State Examination [MMSE] score 26.1 ± 2.4), 120 with probable MCI due to Alzheimer disease (AD) (age 68.6 ± 7.4 years, 45.8% female, MMSE score 25.5 ± 2.8), and 30 with unspecified MCI (age 65.4 ± 9.3 years, 30.0% female, MMSE score 27.0 ± 3.0). Fifty-eight individuals with no cognitive decline or evidence of neurodegenerative disease and 121 individuals lacking brain α-syn deposits at the neuropathologic examination were used as controls.

**Results:**

RT-QuIC identified patients with MCI-LB against cognitively unimpaired controls with 95% sensitivity, 97% specificity, and 96% accuracy and showed 98% specificity in neuropathologic controls. The accuracy of the test for MCI-LB was consistent between the 2 cohorts (97.3% vs 93.7%). Thirteen percent of patients with MCI-AD also had a positive test; of note, 44% of them developed 1 core or supportive clinical feature of dementia with Lewy bodies (DLB) at follow-up, suggesting an underlying LB copathology.

**Conclusions:**

These findings indicate that CSF α-syn RT-QuIC is a robust biomarker for prodromal DLB. Further studies are needed to fully explore the added value of the assay to the current research criteria for MCI-LB.

**Classification of Evidence:**

This study provides Class III evidence that CSF α-syn RT-QuIC accurately identifies patients with MCI-LB.

There is an urgent need for early and disease-specific biomarkers for neurodegenerative diseases to enable proper patient care and selection in clinical trials.^1,2^ The recent development of ultrasensitive assays that indirectly reveal minute amounts of misfolded amyloid proteins in CSF, based on a template amplification strategy, has contributed significantly to this goal.^3^ Current evidence indicates that real-time quaking-induced conversion (RT-QuIC) accurately detects misfolded α-synuclein (α-syn) in the CSF of patients with Parkinson disease or dementia with Lewy bodies (DLB) with an overall sensitivity of 95% and a specificity of 98%.^4-7^ Preliminary data also indicate that the CSF of patients with pure autonomic failure and isolated REM sleep behavior disorder (RBD), 2 prodromal syndromes that often evolve to Parkinson disease or DLB, harbors significant α-syn seeding activity.^7^ However, no study has yet specifically explored the diagnostic value of α-syn RT-QuIC in patients with mild cognitive impairment (MCI), representing a common prodromal clinical manifestation of DLB.^8-10^ Indeed, following the strategies implemented for AD, the diagnostic research criteria for DLB have been recently expanded to include patients in the prodromal stage.^11^ Despite this, however, current research criteria for MCI due to Lewy body (LB) disease, in contrast to those for MCI due to Alzheimer disease (AD), do not consider any biofluid molecular markers.^11^ To fill this gap and to examine whether the CSF α-syn RT-QuIC assay accurately identifies LB-related pathology in patients with MCI, we applied the assay to CSF samples from 2 well-characterized cohorts of participants with MCI classified as having probable MCI-LB, MCI-AD, or unspecified MCI according to current criteria.

## Methods

### Primary Research Question

The primary outcome was the evaluation of the diagnostic performance of the α-syn CSF RT-QuIC assay in patients with probable MCI-LB. Secondary outcomes included evaluating α-syn CSF RT-QuIC as a biomarker of Lewy body copathology in MCI-AD and MCI due to other etiologies.

### Standard Protocol Approvals, Registrations, and Patient Consents

All patients or their next of kin provided written informed consent for use of their clinical data. The local Medical Ethics Committees of Amsterdam UMC and Area Vasta Emilia Centro approved the study.

### Participants

**Figure 1:**
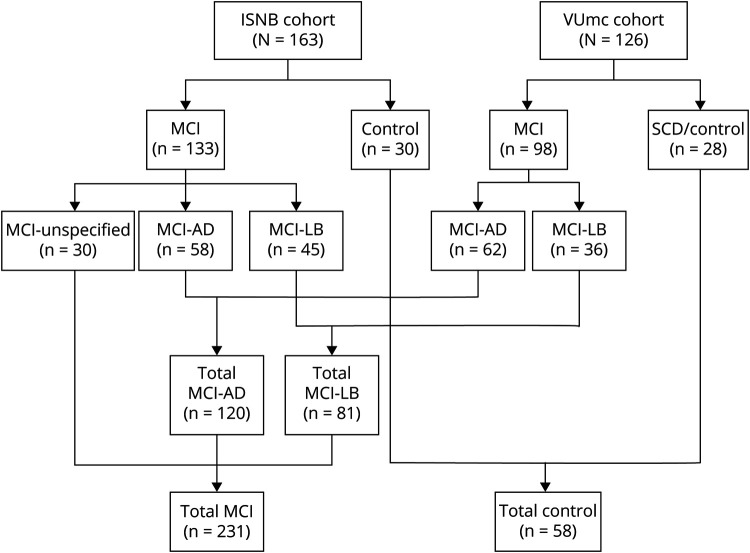
Study Flowchart (Clinical Diagnostic Groups) AD = Alzheimer disease; ISNB = Institute of Neurological Sciences of Bologna; LB = Lewy body; MCI = mild cognitive impairment; SCD = subjective cognitive decline; VUmc = VU Medical Center.

We examined 2 independent cohorts comprising a total of 231 patients with MCI and 58 individuals lacking objective neurologic signs and cognitive decline, herewith defined as controls. The first cohort comprised 163 patients referred to the Institute of Neurological Sciences of Bologna (ISNB), Italy, between 2009 and 2020. The second included 126 individuals referred to the Alzheimer Center Amsterdam, VU Medical Center (VUmc) Amsterdam, the Netherlands, between 2003 and 2020 (figure 1). The ISNB cohort comprised consecutive patients with MCI. The VUmc cohort included patients selected from the Amsterdam Dementia Cohort^12^ and comprised patients with MCI-LB with CSF available, age-matched patients with biomarker-confirmed MCI-AD, and patients with subjective cognitive decline.

In both cohorts, MCI was diagnosed according to current diagnostic criteria, which included concerns regarding changes in cognition, objective impairment in cognition, preservation of the independence of functional abilities, and absence of dementia.^13^

Patients with evidence of nonneurodegenerative causes of cognitive decline, including severe white matter lesions on neuroimaging (Fazekas score 3)^14^ were excluded.

To further evaluate the specificity of the α-syn RT-QuIC assay, we also examined a separate series of 121 CSF samples from individuals referred to the ISNB for a rapidly progressive dementia of various etiologies lacking abnormal α-syn deposits at postmortem CNS neuropathologic examination.

### Clinical Assessment and Classification Criteria of Clinical Diagnostic Groups

We collected clinical history and the results of neurologic examinations and diagnostic investigations. Furthermore, we assessed cognitive function using a standardized battery of neuropsychological tests at both the ISNB^15^ and VUmc.^12^ In both cohorts, parkinsonism was systematically assessed during the neurologic examination and was rated present when the examination showed ≥1 extrapyramidal signs (rest tremor, bradykinesia, and plastic rigidity). At ISNB, cognitive fluctuations, visual hallucinations, and RBD were assessed (present or absent) by a semistructured neuropsychiatric interview and a specific questionnaire on sleep history and complaints. At VUmc, the presence of hallucinations was evaluated with the informant-rated Neuropsychiatric Inventory and scored as being present (Neuropsychiatric Inventory hallucinations score ≥1) or absent. For the MCI-LB cases, the presence or absence of cognitive fluctuations and RBD was based on semistructured interviews rated by 2 independent reviewers.

Diagnostic investigations included brain MRI and, in some cases, ^129^I-ioflupane SPECT (DaTscan) (n = 46), cardiac ^123^I-metaiodobenzylguanidine scintigraphy (n = 11), and polysomnography (n = 25). After CSF collection, most patients (72.2%) were followed up longitudinally (median for the whole patient population 20.3 months, minimum 0 months, maximum 120 months, interquartile range 5–48 months). At VUmc, patients were followed up annually with clinical evaluation (history and neurologic examination), including neuropsychological testing. At the ISNB, follow-up evaluations were carried out by outpatient neurologic visits at the Center for Cognitive Disorders and included serial neuropsychological evaluations in a subgroup of patients (43%).

We used clinical features, AD core markers, imaging, neurophysiologic data, and evolution at the last follow-up to classify the patients into 4 groups: (1) MCI-LB, (2) MCI-AD, (3) MCI due to other neurodegenerative disorders (unsp-MCI), and (4) controls. The presence or absence of clinical core features of DLB was determined according to the definitions and guidelines provided by the DLB Consortium.^11,16^ We also rigorously applied the 1-year rule^11^ to exclude patients with MCI due to Parkinson disease from the studied cohort. The MCI-LB group included 81 individuals (ISNB n = 45, VUmc n = 36) who fulfilled the current criteria for probable MCI-LB^11^ at lumbar puncture (LP; n = 77) or during follow-up (n = 4: 2 from VUmc, 2 from ISNB). Among them, 3 had possible MCI-LB and 1 had unsp-MCI at baseline (at LP).

The MCI-AD group included 120 patients (ISNB n = 58, VUmc n = 62) who lacked clinical evidence of DLB core features at the time of LP and showed in vivo evidence of AD pathology as defined by abnormally reduced β-amyloid_1-42_ (Aβ_42_):β-amyloid_1-40_ (Aβ_40_) ratio (ISNB) or decreased Aβ_42_ levels (VUmc), combined with increased total (t-) tau and phospho (p-) tau concentrations (A+, T+, N+) in CSF or an abnormal p-tau:Aβ_42_ or t-tau:Aβ_42_ ratio.^17-19^

Thirty individuals who did not fulfill the inclusion criteria for MCI-LB (absence of core clinical features and biomarker evidence of DLB at LP and during follow-up) and lacked in vivo evidence of AD pathology by CSF analysis were classified as having unsp-MCI (all from ISNB).

Patients with MCI who progressed to dementia during follow-up received 1 of the following clinical diagnoses according to current criteria: DLB,^16^ AD,^17^ the behavioral variant of frontotemporal dementia,^20^ primary progressive aphasia,^21^ or vascular dementia.^22^

The clinical control group included 30 individuals from the ISNB cohort with a clinical diagnosis of chronic headache or narcolepsy type 1 and no clinical evidence of an underlying progressive neurodegenerative disorder, and 28 individuals from the VUmc cohort who reported subjective experience of cognitive decline but had normal baseline cognition, defined by results of cognitive assessment within normal ranges.^23^ Furthermore, they had at least 1 follow-up assessment (>8 months from baseline) with an unchanged diagnosis.^23^ None of the patients in the clinical groups underwent a postmortem neuropathologic examination.

### CSF Collection and AD Core Marker Analysis

CSF was collected at the time of MCI diagnosis in all patient groups. CSF was obtained by LP following a standard procedure at both centers. Part of the CSF was processed for routine AD biomarker analysis, and the remainder was divided into aliquots and stored according to international CSF biobanking procedures^24^ until RT-QuIC analysis.

At ISNB, CSF t-tau, p-tau, Aβ_42_, and Aβ_40_ were measured by automated chemiluminescent enzyme immunoassay on the Lumipulse G600 platform (Fujirebio, Ghent, Belgium). The Aβ_42_:Aβ_40_ ratio was calculated as previously described.^25^ At VUmc, CSF Aβ_42_, p-tau, and t-tau concentrations were determined with Innotest ELISA (Fujirebio) or Elecsys assays (Roche Diagnostics, GmbH, Mannheim, Germany) run on the Cobas e601 analyzer (Roche Diagnostics, Basel, Switzerland).^26^ Pathologic values for the AD core markers were determined according to internally validated cutoff values at both centers.^26-28^

### Neuropathologic Studies

In the control group with rapidly progressive dementia and postmortem examination, the neuropathologic assessment was performed at the ISNB using standardized procedures as previously described.^7^ To assess neurodegenerative proteinopathies, we performed immunostaining using antibodies against α-syn (clone LB509, working dilution 1:100, Thermo Fisher Scientific, Waltham, MA; and clone KM51, working dilution 1:500, Novocastra, Newcastle Upon Tyne, UK), p-tau (clone AT8, working dilution 1:100, Innogenetics, Ghent, Belgium), A-beta protein (clone 4G8, working dilution 1:5,000, Signet Laboratories, San Francisco, CA), and prion protein (3F4, dilution 1:400, Signet Laboratories) in several brain regions according to established consensus criteria.^7^ Results obtained for 101 of the 121 individuals in this group have been published.^7^

### α-Syn RT-QuIC Assay

We performed the α-syn RT-QuIC assay, including purification of recombinant wild-type human α-syn, as previously described.^7^ To optimize the comparison between the fluorescent responses obtained in different plates, we ran the same positive control throughout all experiments. Furthermore, to minimize possible batch-to-batch variations in α-syn activity and plate-to-plate experimental variability, we normalized the relative fluorescent units at each time point according to the fluorescence peak reached by the positive control and expressed the values as percentages. Each sample was loaded 4 times and considered positive when at least 2 of 4 replicates exceeded the threshold. The latter was calculated by averaging the normalized fluorescence values of negative control replicates during the first 10 hours of all runs plus 30 SDs. The cutoff was set at 30 hours. When only 1 replicate crossed the threshold, the analysis was considered unclear and repeated up to 3 times. All RT-QuIC experiments were performed at the ISNB by personnel blinded to clinical diagnostic groups.

### Statistical Analysis

We used the GraphPad Prism 8.4 software (La Jolla, CA) to analyze and plot the RT-QuIC relative fluorescence responses. The maximum intensity of fluorescence (Imax), lag phase (time required to reach the threshold), and area under the curve (AUC) were extracted for each sample run and statistically analyzed as previously described.^7^ Briefly, depending on the data distribution, the Mann-Whitney *U* test or *t* test (continuous variables) and the χ^2^ test or Fisher exact test (categorical variables) were used, as appropriate, to test for differences between 2 groups. Comparisons between multiple groups were performed with 1-way analysis of variance or Kruskal-Wallis test followed by Tukey or Dunn post hoc test. Pearson and Spearman correlations were performed to test for possible associations between RT-QuIC kinetic parameters and clinical and demographic variables. Statistical significance was set at *p* < 0.05.

To assess the assay performance in discriminating those with MCI-LB from controls and other MCI groups, we calculated the sensitivity, specificity, positive and negative predictive values, and diagnostic accuracy with relative 95% confidence intervals (CIs), first in each study cohort and then in the whole population. To consider the cluster data structure, when evaluating the same discriminatory capacity in the combined cohorts, we applied a mixed-effects logistic regression model with the cohorts as the group variable. The likelihood ratio test revealed no significant differences between the mixed-effect model and a classic logistic regression model, thereby excluding a significant cluster effect. Finally, because we classified the clinical state of patients with MCI-LB only after follow-up, to rule out potential bias, we also calculated the assay sensitivity in the MCI-LB group including only cases with a probable diagnosis at LP.

### Data Availability

The dataset generated and analyzed in the current study is available from the corresponding author on reasonable request.

## Results

The demographic and clinical characteristics of the 2 cohorts are shown in tables 1 and 2. At last follow-up, 72 patients retained the diagnosis of MCI (26 MCI-LB, 33 MCI-AD, 13 unsp-MCI), while 55 fulfilled the criteria for probable DLB (all from the MCI-LB group), 87 for probable AD (all from the MCI-AD group), 12 for behavioral variant of frontotemporal dementia, and 5 for primary progressive aphasia (all from the unsp-MCI group).

**Table 1:**
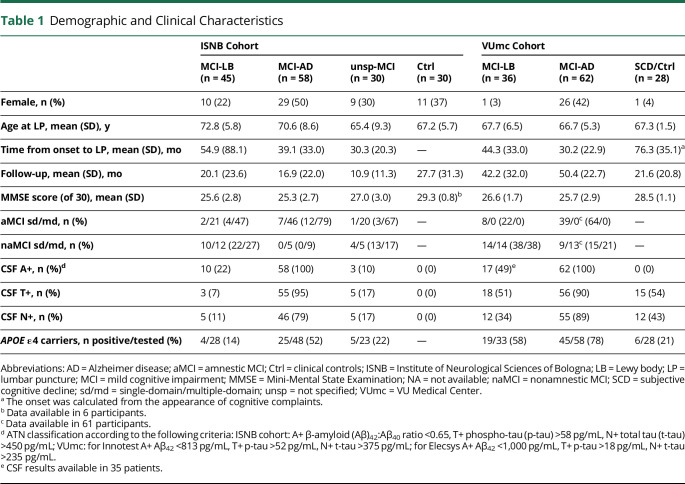
Demographic and Clinical Characteristics

**Table 2:**
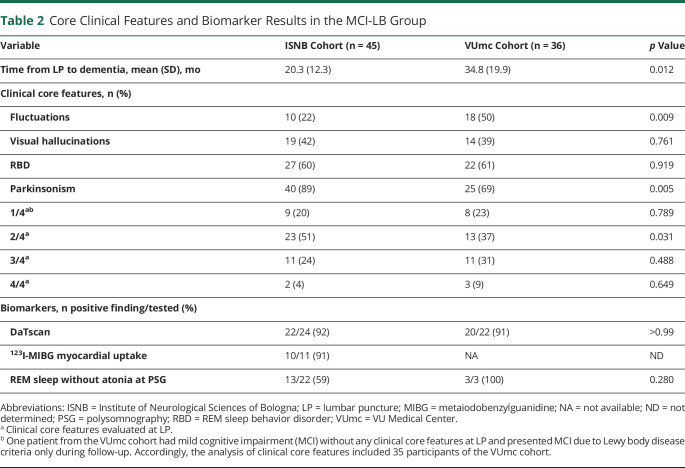
Core Clinical Features and Biomarker Results in the MCI-LB Group

**Figure 2:**
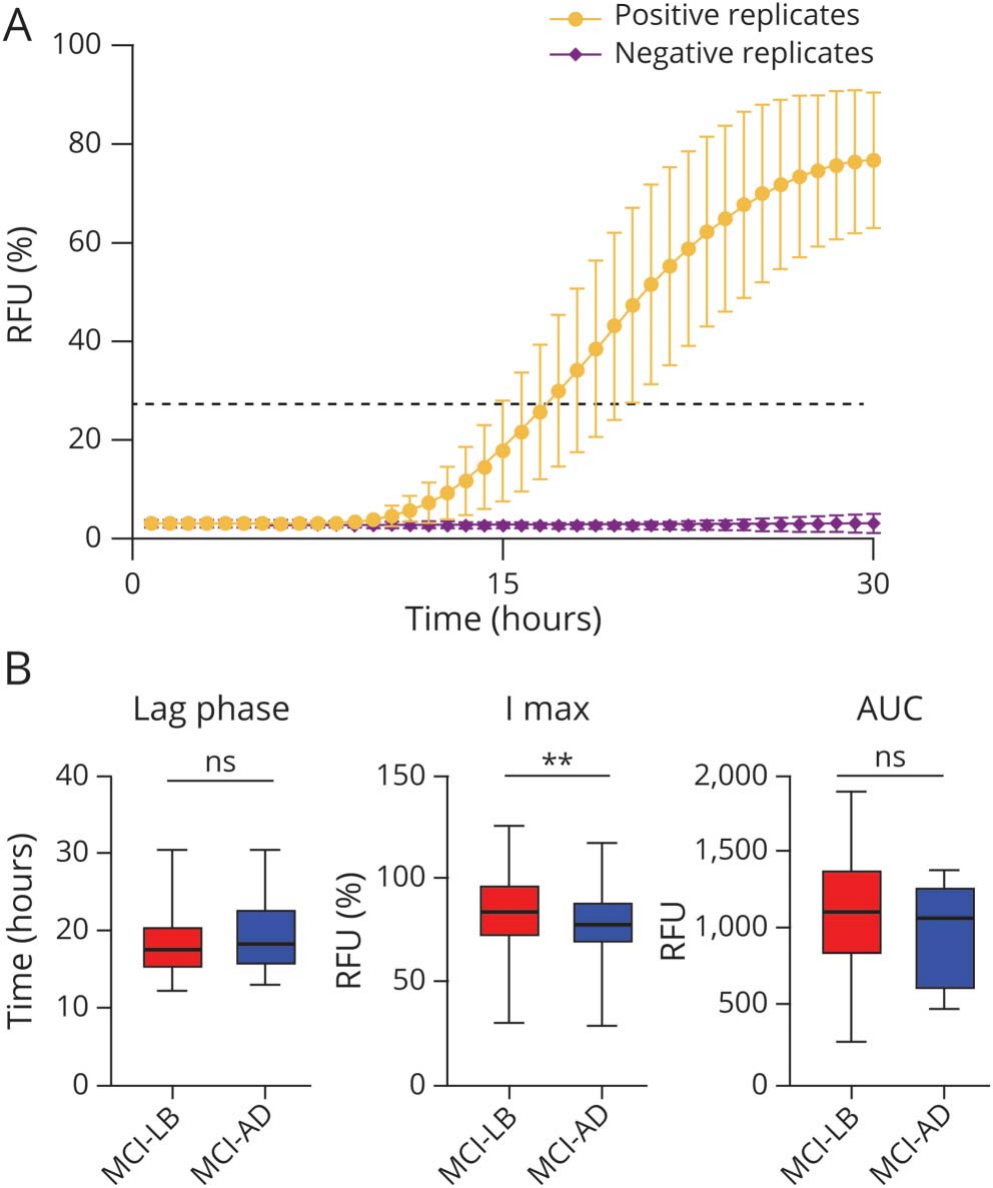
α-Syn RT-QuIC Kinetic Parameters in the Study Cohort (A) Mean normalized fluorescence emission of all α-synuclein (α-syn) real-time quaking-induced conversion (RT-QuIC)–positive cases. Black dashed line represents the threshold. Error bars indicate SD. (B) Comparison of the kinetic parameters of α-syn RT-QuIC–positive cases among the most representative groups (mild cognitive impairment [MCI] due to Lewy body (LB) disease and MCI due to Alzheimer disease [AD]). Statistically significant differences between the 2 groups are limited to the maximum intensity of fluorescence (I max) (**p < 0.01). AUC = area under the curve; ns = nonsignificant; RFU = relative fluorescent units.

Positive α-syn seeding activity was detected in 95.1% of patients with MCI-LB, 13.3% of those with MCI-AD, 6.7% of individuals with unsp-MCI, and 3.4% of controls (table 3 and figure 2A). The relative percentages of positive samples in the MCI-LB and MCI-AD groups were consistent between the ISNB (MCI-LB 97.8% [95% CI 88.2%–99.9%], MCI-AD 12.1% [95% CI 5.0%–23.3%]) and VUmc (MCI-LB 91.7% [95% CI 77.5%–98.3%], MCI-AD 14.6% [95% CI 6.9%–25.8%]) cohorts (table 3). Moreover, in the MCI-LB group, these percentages did not change significantly when patients not fulfilling the diagnostic criteria for probable disease at CSF collection were excluded (ISNB 97.7% [95% CI 87.7%–99.9%], VUmc 91.1% [95% CI 76.3%–98.1%]).

**Table 3:**
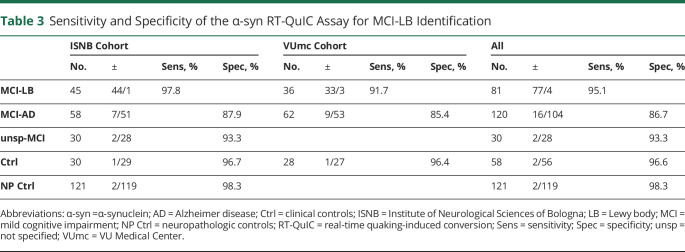
Sensitivity and Specificity of the α-syn RT-QuIC Assay for MCI-LB Identification

Overall, the test showed 95.1% sensitivity (95% CI 87.8%–98.6%), 96.6% specificity (95% CI 88.1%–99.6%), 97.5% positive predictive value (95% CI 90.8%–99.3%), 93.3% negative predictive value (95% CI 84.3%–97.3%), and 95.7% diagnostic accuracy (95% CI 90.8%–98.4%) in the identification of MCI-LB against controls, with only slight differences between the 2 cohorts (table 4). Among the 97 patients with positive α-syn seeding activity, 74 (76.3%) showed a full 4 of 4 positive response, 15 (15.4%) had 3 of 4 positive replicates, and only 8 (8.2%) had 2 of 4 positive replicates.

**Table 4:**
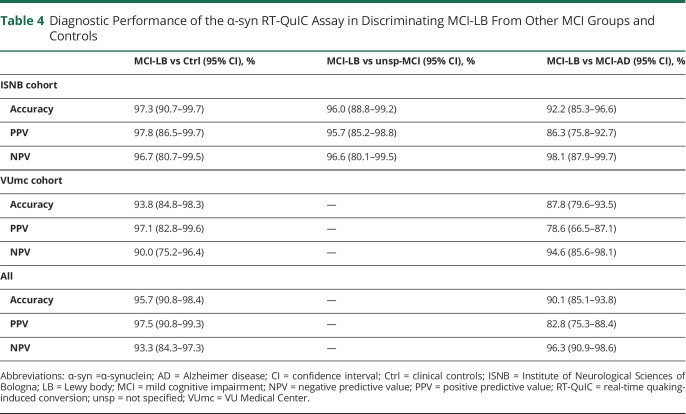
Diagnostic Performance of the α-syn RT-QuIC Assay in Discriminating MCI-LB From Other MCI Groups and Controls

**Figure 3:**
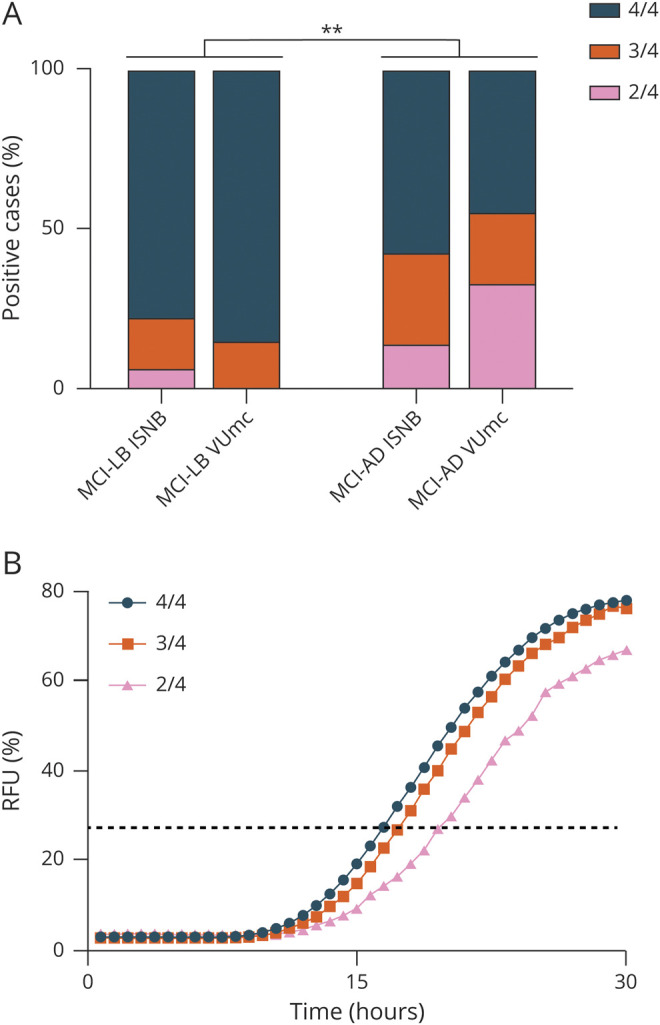
Analysis of Replicate Kinetic Curves and Distribution in the ISNB and VUmc Cohorts (A) Distribution analysis of positive replicates in the mild cognitive impairment (MCI) due to Lewy body (LB) disease and MCI due to Alzheimer disease (AD) cohorts. Statistical analyses by the χ^2^ test resulted in **p < 0.01. (B) Kinetic curves across positive cases showing different α-synuclein real-time quaking-induced conversion responses by distinct numbers of positive replicates. Error bars are omitted to increase the readability of the image. Black dashed line represents the threshold. ISNB = Institute of Neurological Sciences of Bologna; RFU = relative fluorescent units; VUmc = VU Medical Center.

Comparison of the parameters that describe the kinetic curve of the RT-QuIC positive reactions, indicating the degree of seeding activity present in the sample, revealed a statistically significant difference in Imax between the MCI-LB and MCI-AD groups (83.4% [95% CI 81.3–85.5] vs 74.6% [95% CI 69.0–80.3], *p* = 0.002) (figure 2B). Specifically, 3 MCI-AD samples in the VUmc cohort showed a delayed lag phase and reduced amplitude of the fluorescence response. In addition, there was a higher proportion of samples with 2 of 4 positive replicates in the MCI-AD group than in the MCI-LB group (25.0% [95% CI 7.3%–52.4%] vs 3.9% [95% CI 0.8%–10.9%], *p* = 0.015) (figure 3A), which also showed a significantly longer lag phase (21.3 hours [95% CI 19.1–23.5] vs 18.2 hours [95% CI 17.8–18.6], *p* = 0.008) and a lower Imax (70.5% [95% CI 61.2%–79.8%] vs 82.9% [95% CI 80.8%–85.0%], *p* = 0.024) and AUC (755.4 [95% CI 537.5–973.3] vs 1,129.0 [95% CI 1,074.0–1,183.0], *p* = 0.003) compared to those showing a full 4 of 4 response (figure 3B).

In contrast, there were no significant differences in the rate of positivity, the number of positive wells, and kinetics of fluorescent RT-QuIC response between the 5 cases who did not fulfill the MCI-LB clinical criteria at LP and those with probable MCI-LB at LP (data not shown). Similarly, in the MCI-LB group, we did not find any significant correlation between the kinetic parameters of positive RT-QuIC signals (i.e., Imax, lag phase, and AUC) and demographic and clinical variables, including age, time from onset to LP, time from LP to conversion to dementia, Mini-Mental State Examination score, and CSF Aβ status (A+ vs A−) (data not shown).

Three of the 4 patients with probable MCI-LB who had a negative result by α-syn RT-QuIC showed typical DLB clinical features (i.e., presence of at least 2 core clinical criteria), whereas the fourth showed only mild asymmetric bradykinesia associated with a slight abnormality on DaTscan. In that patient, follow-up was limited due to a psychiatric comorbidity, which led to death in a psychiatric hospital 1 year after diagnosis.

Of note, 6 of the 16 patients with MCI-AD who tested positive by α-syn RT-QuIC developed 1 DLB clinical core feature at follow-up (i.e., visual hallucinations in 4, probable RBD in 2), suggesting an underlying LB copathology. Furthermore, 1 additional participant in this group developed orthostatic hypotension, which is a supportive clinical criterion for DLB. In the RT-QuIC–positive AD subgroup, 12 patients were classified as having amnestic MCI (7 multidomain and 5 single domain) and 4 as having nonamnestic (3 multidomain, 1 single domain) MCI. Finally, the 2 patients with unsp-MCI and the 2 individuals from the clinical control group who tested positive by α-syn RT-QuIC did not show any LB-related clinical features either at the first evaluation or at follow-up.

We previously demonstrated the high specificity of our α-syn RT-QuIC assay in 101 individuals lacking LB-related pathology at postmortem examination.^7^ Since then, we have tested 20 CSF samples from neuropathologically verified individuals showing no brain deposition of α-syn. The final diagnoses of these cases included sporadic Creutzfeldt-Jakob disease (n = 12), genetic Creutzfeldt-Jakob disease (n = 3), subcortical arteriosclerotic encephalopathy (n = 2), AD (n = 1), limbic encephalitis (n = 1), and autosomal dominant cerebellar ataxia, deafness, and narcolepsy (n = 1). All CSF samples from these additional individuals showed negative results, yielding an overall specificity of 98.3% (95% CI 94.2%–99.8%) in this cohort of 121 neuropathologic controls.

## Discussion

The results of the present study demonstrate that the CSF α-syn RT-QuIC assay accurately detects LB disease in patients with MCI. Application of the assay in 2 large distinct groups of patients representing the MCI clinical spectrum identified those diagnosed with probable MCI-LB with a 95.1% overall sensitivity. Furthermore, the test demonstrated 96.6% specificity against cognitively unimpaired controls and close to perfect (98.3%) specificity for LB-related pathology in a cohort of 121 pathologic controls lacking LB at postmortem examination. Five of the examined patients with MCI-LB did not fulfill the requirements for probable disease at LP yet demonstrated a full positive α-syn RT-QuIC response. Moreover, the significant number of positive MCI-LB cases with an amnestic MCI profile suggests that α-syn RT-QuIC assay could provide predictive data on dementia evolution in patients with atypical cognitive DLB profiles. Taken together, these findings are in line with preliminary results obtained in patients with isolated RBD, patients with pure autonomic failure, and those with incidental LB at postmortem examination,^7^ demonstrating that patients with LB harbor significant α-syn seeding activity early in the course of the disease, regardless of clinical presentation. Consequently, the detection of abnormal α-syn species by RT-QuIC not only is very accurate but also represents an early biomarker for LB disease.

The demonstration of abnormal α-syn species in 13.3% of patients diagnosed with probable MCI-AD deserves specific comment. Several factors suggest that the positive α-syn RT-QuIC results in these patients provide evidence of genuine LB copathology. First, the data we obtained from the postmortem-verified cohort strongly indicate that the specificity of the assay for LB-related pathology is very high. Second, 43.7% of individuals with MCI-AD showing α-syn seeding activity developed clinical signs suggestive of DLB. Third, mixed pathology is frequently encountered in DLB, especially concomitant AD pathology. This has been shown in several neuropathologic studies, highlighting the high frequency of copathologies in patients with a clinical diagnosis of AD.^29,30^ In one of the largest cohorts, 177 of 626 (28%) participants with clinical AD collected at the Mayo Clinic Brain Bank from 2007 to 2016 showed significant LB copathology compatible with a secondary diagnosis of DLB.^31^ Given the temporal gap between MCI clinical diagnosis and death, during which copathologies may develop, and the relative rise of DLB diagnosis due to the refinement of clinical criteria,^11,16^ the 13.3% figure we obtained appears reasonable.

Additionally, the finding of occasional positive samples in individuals with unsp-MCI, those with subjective cognitive decline, or other clinical controls is also consistent with the notion that incidental LB pathology affects 5% to 8% of people >60 years old in the absence of extrapyramidal signs or cognitive decline.^32,33^

We do not have a definite explanation for the negative results obtained in a few patients with MCI-LB. At least 2 of them had typical features and developed probable DLB at follow-up. Blood contamination, the only preanalytical variable with a proven negative effect on RT-QuIC performance, could be excluded by visual inspection. However, RT-QuIC is a relatively new technique, and additional studies are needed to fully address the effect of preanalytical variables and potential CSF contaminants or tube absorption on assay performance. The existence of rare disease subtypes associated with a distinct molecular pathology, possibly determined by genetic factors, as previously shown in Parkinson disease,^3^ might be an alternative explanation. Finally, a minority of false-positive clinical diagnoses of DLB, not sustained by LB pathology, can be expected, especially in patients with AD.

The development of cell-free protein aggregation assays such as RT-QuIC originated from the discovery of the protein-only mechanism of prion propagation.^3^ The first version of the RT-QuIC assay was developed to detect pathogenic prion protein seeds in CSF.^34,35^ After the recognition of the high accuracy of the prion RT-QuIC in identifying misfolded forms of prion protein in the CSF, the clinical diagnostic criteria for sporadic Creutzfeldt-Jakob disease, the most common human prion disease, were significantly revised in 2017 to accommodate the novel biomarker.^36^ Most significant, previous stringent clinical criteria introduced to increase specificity, given the lack of an accurate biomarker, were dismissed. Thus, any progressive neurologic syndrome resulting in a positive prion RT-QuIC test currently receives a diagnosis of probable sporadic Creutzfeldt-Jakob disease. Given the 5-year gap between the introduction of the prion and α-syn RT-QuIC assays, it is foreseeable that the impact of the latter on diagnostic criteria, and ultimately in clinical practice, will follow the same course. To this end, the present results must be validated in an interlaboratory setting and confirmed in larger cohorts including less stringent clinical selection criteria (e.g., possible MCI-LB or even MCI with risk factors for DLB) and long follow-up or postmortem confirmation to fully establish the clinical predictive value of α-syn RT-QuIC and its added value compared to current criteria and biomarkers The validation of α-syn oligomer detection by RT-QuIC or other seeding assays as pathology-driven biomarkers of LB disease might lead to the progressive simplification of current stringent clinical selection criteria for MCI-LB and the proposal of diagnostic categories allowing biomarker-based evidence of co-occurring pathologies (e.g., MCI-AD-LB).

The urgent need for specific biomarkers concerns not only the early diagnosis and clinical management of patients but also the design and outcome of clinical trials. In addition to searching for novel therapies against α-syn pathology in LB disease, the availability of an accurate early biomarker may improve patient selection in AD trials.^37^ Indeed, imperfect patient selection is considered a possible cause of therapeutic trial failures in neurodegenerative dementias due to frequently occurring copathologies.^38,39^

A significant limitation of the present study is the lack of postmortem data for the examined MCI cohorts. However, the selection of well-characterized patients fulfilling the criteria for probable disease has likely significantly limited the discrepancy between clinical and postmortem diagnoses in the most relevant MCI-LB group. In this regard, it is also relevant that a substantial portion of the patients with MCI-LB in both cohorts had a positive DaTscan, which showed a significant correlation with LB pathology in postmortem studies.^40,41^ Additional limitations include the fact that we did not collect the indicative biomarkers^11^ uniformly but only in a subgroup of patients with MCI-LB and that the average clinical follow-up period was rather short in the ISNB cohort. Moreover, the in vivo control group we used did not include healthy controls but patients without cognitive impairment or signs/symptoms of neurodegenerative disease or patients with subjective symptoms, which is a condition associated with a higher risk of AD. Finally, the facts that control groups were not equally represented in the study sets and that age matching did not refer to individual groups may have introduced some biases.

We showed that the detection of abnormal α-syn species by RT-QuIC represents a valuable biomarker for the in vivo demonstration of LB pathology in patients with MCI. This finding is highly relevant given the lack of a biofluid disease-specific marker for LB pathology in current diagnostic criteria for this prevalent clinical presentation. The novel biomarker accurately identified patients with DLB at the prodromal clinical stage and demonstrated high specificity in a large cohort of individuals examined neuropathologically. Thus, its implementation after validation may help the clinical management and recruitment for clinical trials in memory clinics.

## Glossary

α-syn: α-synuclein
Aβ_40_: β-amyloid_1-40_
Aβ_42_: β-amyloid_1-42_
AD: Alzheimer disease
AUC: area under the curve
CI: confidence interval
DLB: dementia with Lewy bodies
Imax: maximum intensity of fluorescence
ISNB: Institute of Neurological Sciences of Bologna
LB: Lewy body
LP: lumbar puncture
MCI: mild cognitive impairment
MMSE: Mini-Mental State Examination
p-tau: phospho-tau
RBD: REM sleep behavior disorder
RT-QuIC: real-time quaking-induced conversion
t-tau: total tau
unsp: unspecified
VUmc: VU Medical Center
